# Potential of High-Affinity, Slow Off-Rate Modified Aptamer Reagents for Mycobacterium tuberculosis Proteins as Tools for Infection Models and Diagnostic Applications

**DOI:** 10.1128/JCM.00469-17

**Published:** 2017-09-25

**Authors:** Theresa M. Russell, Louis S. Green, Taylor Rice, Nicole A. Kruh-Garcia, Karen Dobos, Mary A. De Groote, Thomas Hraha, David G. Sterling, Nebojsa Janjic, Urs A. Ochsner

**Affiliations:** a SomaLogic, Inc., Boulder, Colorado, USA; b Department of Microbiology, Immunology & Pathology, Colorado State University, Fort Collins, Colorado, USA; Carter BloodCare & Baylor University Medical Center

**Keywords:** Mycobacterium tuberculosis, aptamer, biomarker, immunodiagnostics, proteomics

## Abstract

Direct pathogen detection in blood to diagnose active tuberculosis (TB) has been difficult due to low levels of circulating antigens or due to the lack of specific, high-affinity binding reagents and reliable assays with adequate sensitivity. We sought to determine whether slow off-rate modified aptamer (SOMAmer) reagents with subnanomolar affinity for Mycobacterium tuberculosis proteins (antigens 85A, 85B, 85C, GroES, GroEL2, DnaK, CFP10, KAD, CFP2, RplL, and Tpx) could be useful to diagnose tuberculosis. When incorporated into the multiplexed, array-based proteomic SOMAscan assay, limits of detection reached the subpicomolar range in 40% serum. Binding to native M. tuberculosis proteins was confirmed by using M. tuberculosis culture filtrate proteins and fractions from infected macrophages and via affinity capture assays and subsequent mass spectrometry. Comparison of serum from culture-positive pulmonary TB patients and TB suspects systematically ruled out for TB revealed small but statistically significant (*P* < 0.0001) differences in the median M. tuberculosis signals and in specific pathogen markers, such as antigen 85B. Samples where many M. tuberculosis aptamers produced high signals were rare exceptions. In concentrated, protein-normalized urine from TB patients and non-TB controls, the CFP10 (EsxB) SOMAmer yielded the most significant differential signals (*P* < 0.0276), particularly in TB patients with HIV coinfection. In conclusion, direct M. tuberculosis antigen detection proved difficult even with a sensitive method such as SOMAscan, likely due to their very low, subpicomolar abundance. The observed differences between cases and controls had limited diagnostic utility in serum and urine, but further evaluation of M. tuberculosis SOMAmers using other platforms and sample types is warranted.

## INTRODUCTION

Tuberculosis (TB) remains a major global health problem, and in 2015 it had the highest mortality of any infectious disease worldwide. While there has been a steady yet slow decline in new TB cases at a rate of 2% per year recently, incidence remains high in Africa, particularly in sub-Saharan Africa, Asia, the Western Pacific Region, and in Central and South America, totaling 10.4 million new TB cases in the world in 2015 (1). TB mortality rate has decreased by almost half since 1990, but there are still over a million deaths per year, with about one-fourth of these deaths occurring among people with HIV.

An estimated 4.3 million new TB cases a year remain undiagnosed (1, 2). Since pulmonary TB represents the majority of TB cases and is transmitted via aerosols from people with active pulmonary disease, a high diagnostic priority is to determine those with active TB to enable rapid treatment and reduce disease transmission. High-priority diagnostic needs for which specific target product profiles (TPPs) have been defined include a non-sputum-based biomarker test for all forms of TB and a simple, low-cost triage test for use by first-contact care providers as a rule-out test (3–5). Traditional methods for pathogen detection are culture and/or staining microscopy of the Mycobacterium tuberculosis bacilli. Several classes of M. tuberculosis-specific pathogen products are potential candidates for new diagnostic methods, including lipoarabinomannan (LAM), metabolites (including lipids, sugars, and volatile organic compounds in breath), nucleic acids, peptides, and proteins (6, 7). Sputum smear microscopy for TB diagnosis is still widely used around the world and is fast and inexpensive, although it is suboptimal in children and in people with HIV (8, 9). Culture-based diagnostic methods are more sensitive but require several weeks to obtain results (10), which causes a delay in therapy. The tuberculin skin test (TST) and the gamma interferon release assay (IGRA) measure the immune response to M. tuberculosis antigens and thus do not distinguish active TB from latent disease or a previously cleared infection (11, 12). Gene-Xpert MTB/RIF is a rapid sputum molecular diagnostic test which has been rolled out in many countries and performs very well, except perhaps in smear-negative TB and pediatric TB cases (13–17). Gene-Xpert has transformed TB diagnostics, although it requires complex and expensive cartridges and a reliable power source. Alternative rapid, accurate tests for point-of-care TB diagnostics using non-sputum-based samples and new or improved technologies are critically needed (6).

Our proteomic technology is based on affinity-binding reagents and a technology platform targeting proteins that are intact or, at a minimum, harbor the native structural epitopes used for selection of the binding reagents. SOMAmers are a new class of synthetic reagents, in some ways similar to monoclonal antibodies, and are used in proteomic applications where high sensitivity and specificity is needed. Advantages of SOMAmers over antibodies include higher multiplexing capabilities due to low cross-reactivity and universal assay conditions, chemical stability to heat, drying, and solvents, reversible renaturation, ease of reagent manufacturing, consistent lot-to-lot performance, and lower cost. Modified aptamers are the basis for the SOMAscan multiplex proteomic assay that measures thousands of proteins simultaneously with high precision (<5% coefficient of variation) in a small sample volume of <150 μl. The overall dynamic range of the assay is roughly 8 logs, with a median lower limit of detection of 40 fM (18, 19).

We initiated a broad TB biomarker discovery effort using serum and urine samples from initial TB suspects that had confirmed TB or were ruled out for TB (non-TB, or NTB) based on protocolized culture and systematic follow-up. In this report, we focus on the generation of specific high-affinity binding reagents to M. tuberculosis pathogen-derived proteins and evaluate their utility for direct M. tuberculosis antigen detection. A separate, accompanying report by De Groote et al. describes the identification of host response markers and the performance of a TB-specific biosignature entirely based on host markers (20).

A multitude of M. tuberculosis proteins may be useful as potential diagnostic TB targets, given the observed immunologic responses in TB patients stemming from serology studies or infection models (21–24). We chose 18 mycobacterial proteins for M. tuberculosis SOMAmer development via systematic evolution of ligands by exponential enrichment (SELEX) (19, 25). M. tuberculosis protein targets included extracellular, cell surface-associated, and intracellular factors that may be circulating in TB patients and may be detectable with a highly sensitive and specific assay. FbpA, FbpB, and FpbC form the antigen 85 complex, are highly abundant mycolyltransferases essential for cell wall synthesis, and are also major secretory antigens (26, 27). ESAT-6 (EsxA) and CFP10 (EsxB) represent excellent diagnostic targets, since they are absent from Mycobacterium bovis BCG (28). PstS1 is a Mycobacterium-specific lipoprotein phosphate transporter (29). MPT64 and MPT51 are major extracellular antigens and once were considered to improve the BCG vaccine (30, 31). α-Crystalline (Acr, HspX) is an abundant inner membrane protein induced by microaerobic and anoxic conditions and plays a role in long-term viability during latent infection (32). CFP30 and MTB12 (CFP-2) are found in culture supernatants and are known antigens during infection (33, 34). Other targets described as potential diagnostic markers were GroES (CH10), GroEL2 (CH602), DnaK, the 50S ribosomal protein RplL, Adk (KAD), MasZ, and Tpx (35–39). These intracellular proteins are produced constitutively and at high levels, although these factors are less specific for M. tuberculosis, since closely related proteins are found in nontuberculous mycobacteria (NTM) and other actinobacteria, such as Nocardia and Streptomyces.

Here, we describe the characterization of the pathogen-specific aptamers and the identification of those with the greatest sensitivity and specificity against recombinant M. tuberculosis proteins, natively expressed M. tuberculosis culture filtrate proteins, and fractions from M. tuberculosis-infected human macrophages. Finally, the top-performing M. tuberculosis aptamers were examined as potential diagnostic tools in well-curated clinical samples.

## RESULTS

### M. tuberculosis SOMAmer development and characterization.

SOMAmer reagents were generated for 18 M. tuberculosis targets (see Table S2 in the supplemental material), and subsequent characterization of the binding agents and their performance in a variety of direct antigen detection assays produced a list of the top 19 aptamers for 10 different M. tuberculosis targets (Table 1). In many cases, a multitude of sequences with different modified nucleotides but similar binding properties and affinities were obtained. Among the targets yielding SOMAmers with the best affinity were the antigen 85 proteins A85A (Rv3804c), A85B (Rv1886c), and A85C (Rv0129c). Some of the binding agents showed strong cross-reactivity between the three antigen 85 proteins (Table S3), which was not surprising given the high degree of structural and amino acid sequence identity (67 to 79%). Still, antigen 85 binding reagents with low cross-reactivity or nearly monospecific activity were also obtained. Since antigen 85 proteins contain motifs that interact with fibronectin and such complexes are thought to form during TB infection (40), we focused on SOMAmers that can bind antigen 85 protein both in free form and in a complex with fibronectin (Table S3).

**TABLE 1:** High-affinity SOMAmer binding reagents for M. tuberculosis proteins

Target (gene)	Function or name(s)	Molecular mass (kDa), native	pI	SOMAmer	Modified nucleotide	*K_D_*^*a*^ (nM)	*K_D_*, app^*b*^ (nM)
A85A (Rv3804c)	Mycolyltransferase, antigen 85A, FbpA	31.7	5.32	14504-6	PPdU	0.07	0.003
A85B (Rv1886c)	Mycolyltransferase, antigen 85B, FbpB	30.7	4.87	12074-11	NapdU	0.14	0.002
				12074-5	NapdU	0.08	0.002
A85C (Rv0129c)	Mycolyltransferase, antigen 85C, FbpC	32.1	4.99	14494-124	2NapdU	0.02	0.001
				14506-48	PPdU	0.04	0.017
				4950-27	NapdU	0.03	0.002
				5569-2	2NapdU	0.01	0.005
CH10 (Rv3418c)	10-kDa chaperonin, GroES	10.8	4.62	14488-1	2NapdU	0.53	0.005
CH602 (Rv0440)	60-kDa chaperonin 2, GroEL2	56.7	4.85	14484-4	2NapdU	0.50	0.010
				7592-57	2NapdU	0.46	0.017
DNAK (Rv0350)	Chaperone, Hsp70	66.8	4.85	14486-2	2NapdU	1.03	0.007
				7606-49	TrpdU	0.66	0.039
ESXB (Rv3874)	ESAT-6-like protein EsxB, CFP10	10.8	4.59	5557-2	2NapdU	0.54	0.004
KAD (Rv0733)	Adenylate kinase	20.1	5.02	14491-10	2NapdU	0.08	0.001
				14491-43	2NapdU	0.12	0.001
MTB12 (Rv2376c)	Low-molecular-wt antigen, CFP-2	16.6	5.10	15001-182	NapdU	0.05	0.004
				15001-2	NapdU	0.04	0.003
RL7 (Rv0652)	50S ribosomal protein L7/L12, RplL	13.4	4.59	7587-49	TrpdU	0.07	0.002
				7596-2	2NapdU	0.13	0.001

a Radiolabel equilibration binding assay.

b SOMAscan assay.

Other targets for which SOMAmer reagents with subnanomolar affinity were obtained included CH10 (Rv3418c), CH602 (Rv0440), DNAK (Rv0350), ESXB (Rv3874), KAD (Rv0733), MTB12 (Rv2376c), and RL7 (Rv0652).

Challenge with human serum during SELEX to counterselect the DNA libraries for aptamers with undesired nonspecific binding to serum proteins proved to be efficient, as shown by a 1.9- to 7.3-fold lower background signal compared to those of SOMAmers from standard SELEX without serum counterselection and, in most cases (e.g., for MPT64), improved affinity (Table S4).

### SOMApanel validation of M. tuberculosis SOMAmers in serum and urine.

**FIG 1:**
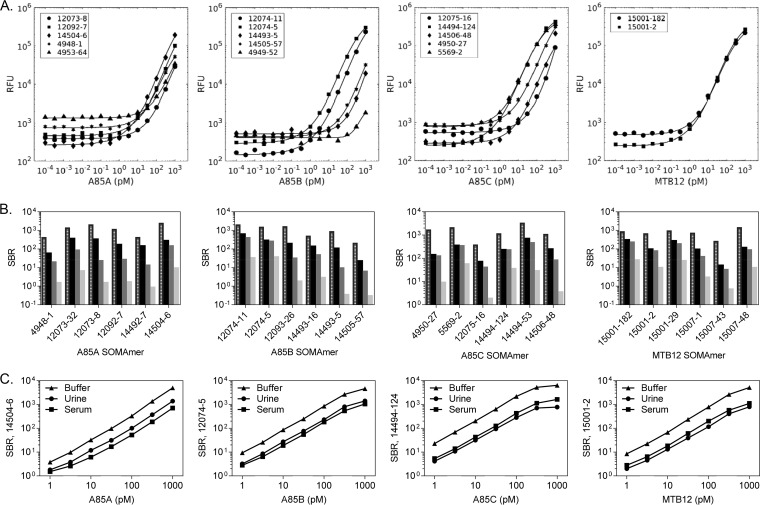
Characterization of M. tuberculosis SOMAmers. (A) SOMAmer signal responses to their recombinant targets titrated into 40% human serum. TB SOMApanel data are shown for different SOMAmer reagents selected with A85A, A85B, A85C, and MTB12. (B) Determination of signal-to-background ratios (SBRs) to identify the most sensitive and specific M. tuberculosis aptamers for antigen 85 complex proteins and MTB12 proteins. Buffer or serum (40%) was spiked with recombinant proteins: buffer, 5 nM (hatched bars); serum, 5 nM (black bars); serum, 0.5 nM (dark gray bars); serum, 0.05 nM (light gray bars). (C) Comparison of SBRs for 4 M. tuberculosis reagents demonstrating SOMAmer-dependent and matrix-dependent responses.

The relative performance of the M. tuberculosis SOMAmers was compared using a custom, targeted version of SOMAscan (TB SOMApanel), which contained probes for 86 M. tuberculosis aptamers targeting 18 M. tuberculosis protein targets. Data specific for M. tuberculosis antigen 85 complex (A85A, A85B, and A85C) and MTB12 proteins are presented below (Fig. 1), and summarized data for all 86 M. tuberculosis SOMAmers are provided in Table S2.

Serum and urine are diagnostic specimens of interest; therefore, we evaluated the ability of the binding agents to detect recombinant M. tuberculosis proteins in these complex matrices. Relative fluorescence unit (RFU) signals in response to 16-point titrations in 40% human serum revealed differences in sensitivity and specificity of the M. tuberculosis aptamers, particularly for A85B (Fig. 1A). All aptamers performed well in buffer but differed significantly in their performance in serum. The specificity of SOMAmers was apparent by the lower RFU plateau (*y* axis), which is affected by off-target interactions with human proteins in serum and defined the background level for each reagent. Increased sensitivity was evident by both a left shift of the titration curves toward lower protein concentrations (*x* axis) and a low background plateau. Based on these analyses, SOMAmers 12074-11 and 12074-5 were the most sensitive and specific A85B aptamers.

Signal-to-background ratios (SBRs) were determined for the M. tuberculosis aptamers to provide a quantitative measure of sensitivity and specificity. SBR data for 32 M. tuberculosis aptamers detecting A85A, A85B, A85C, and MTB12 proteins titrated in 40% serum indicated that many M. tuberculosis SOMAmers yielded protein spike-dependent signals several orders of magnitude above background signals, even at subnanomolar protein spike concentrations (Fig. 1B).

Pooled human urine was also examined as a sample matrix, and the urine was concentrated to allow for interrogation of a known amount of matrix protein. Comparisons of SBRs for 4 M. tuberculosis SOMAmers from recombinant protein titrations in urine protein concentrates (100 μg), serum (40%), and buffer were performed (Fig. 1C). Reagent performance was matrix dependent, and all M. tuberculosis aptamers yielded higher SBRs in protein-free buffer due to the elimination of off-target interactions. There were differences in SOMAmer performance in the protein-rich and complex matrices of serum and urine concentrates. For example, the A85A and A85B aptamers performed better in urine concentrates than in serum. The opposite trend was observed for the A85C and MTB12 SOMAmers. Such protein titrations were used to determine the limits of detection (LOD) and quantitation (LOQ) of M. tuberculosis aptamers for their recombinant protein targets in the SOMApanel assay and in each matrix type (Table 2).

**TABLE 2:** LOD and LOQ for 21 top-performing M. tuberculosis SOMAmers in buffer, serum, and urine protein concentrates^*a*^

Target; SOMAmer	LOD (pM) and LOQ (pM) in:						Serum bacterial load (LOD; cells/ml)
	Buffer (SB17T)		Normal serum (40%)		Urine protein (100 μg)		
	LOD	LOQ	LOD	LOQ	LOD	LOQ	
A85A; 14504-6	0.29	0.74	0.97	2.44	0.92	2.25	7.3 × 10^6^
A85B; 12074-11	0.36	0.82	0.72	1.56	0.18	1.30	1.8 × 10^6^
A85B; 12074-5	0.27	0.60	0.18	0.47	0.30	1.16	5.5 × 10^5^
A85B; 14505-57	4.15	5.83	5.13	13.66	55.47	62.67	4.0 × 10^7^
A85C; 12075-16	7.39	8.40	5.38	15.58	8.51	13.88	4.9 × 10^8^
A85C; 14494-124	0.14	0.47	0.07	0.22	0.06	0.23	2.7 × 10^7^
A85C; 14506-48	0.89	2.13	1.98	3.84	2.10	6.15	3.3 × 10^8^
A85C; 4950-27	0.36	0.70	1.80	3.58	0.61	1.88	3.4 × 10^8^
A85C; 5569-2	0.23	0.58	0.28	0.68	0.36	0.96	2.6 × 10^7^
CH10; 14488-1	0.40	1.35	0.84	3.25	1.94	5.94	1.1 × 10^6^
CH602; 14484-4	0.34	0.65	0.56	2.50	1.20	5.72	2.2 × 10^8^
CH602; 7592-57	0.26	0.72	1.47	4.76	1.98	7.02	3.0 × 10^7^
DNAK; 14486-2	0.31	0.77	0.78	2.41	3.03	6.01	7.4 × 10^6^
DNAK; 7606-49	0.59	1.52	1.53	7.10	2.19	8.72	1.6 × 10^7^
ESXB; 5557-2	0.12	0.38	0.32	0.77	0.13	0.34	2.2 × 10^6^
KAD; 14491-10	0.20	0.62	1.11	3.36	24.55	47.99	7.9 × 10^7^
KAD; 14491-43	0.14	0.55	0.34	1.35	6.19	21.20	3.5 × 10^7^
MTB12; 15001-182	0.18	0.62	1.68	2.48	0.93	1.82	3.4 × 10^5^
MTB12; 15001-2	0.18	0.57	0.11	0.38	0.42	1.72	1.1 × 10^6^
RL7; 7587-49	0.26	0.93	2.47	8.08	1.05	14.01	1.2 × 10^8^
RL7; 7596-2	0.08	0.22	0.70	1.67	1.35	7.34	3.4 × 10^7^

a LOD and LOQ were determined by SOMApanel assay of recombinant protein spike titrations. Also shown are the bacterial loads estimated from the LOD of native CFP titrations in serum.

### M. tuberculosis SOMAmer validation with native M. tuberculosis proteins.

M. tuberculosis aptamers were raised against recombinant pathogen proteins derived from sequences from M. tuberculosis strain H37Rv. To examine M. tuberculosis SOMAmer responses to natively generated M. tuberculosis H37Rv proteins, proteins produced by the pathogen during the infection of cultured human macrophages (macrophage lysates) and those secreted by the pathogen during *in vitro* cultivation (culture filtrate proteins, or CFPs) were also examined.

**FIG 2:**
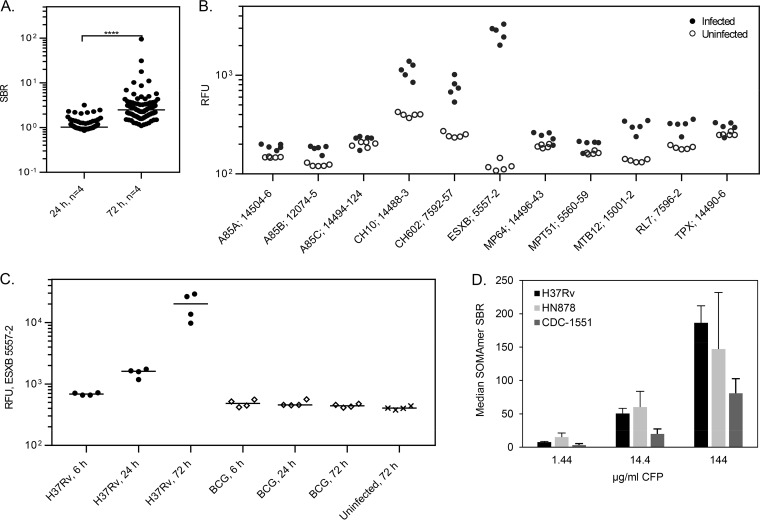
SOMAmer validation with native M. tuberculosis proteins. (A) SBRs for 86 M. tuberculosis aptamers at 24-h and 72-h infection time points in M. tuberculosis-infected human macrophage lysates *in vitro*. (B) Shift of M. tuberculosis SOMAmer signals in M. tuberculosis-infected macrophages lysates compared to uninfected controls at 72 h. (C) Time course study showing signals from M. tuberculosis aptamer ESXB 5557-2 in H37Rv-infected (●), BCG-infected (◇), or uninfected human macrophages (×). (D) Median SBR of 86 M. tuberculosis SOMAmers in response to CFPs from three M. tuberculosis strains (H37Rv, HN878, and CDC-1551) and at three protein levels (1.4, 14.4, and 144 μg/ml) in buffer.

SBRs demonstrated that M. tuberculosis SOMAmers detected target proteins within M. tuberculosis-infected macrophage lysates, and many signals increased with duration of infection (Fig. 2A). By the 72-h time point, numerous M. tuberculosis aptamers detected their natively expressed targets within the macrophage lysates, with the most dramatic increases observed from CFP10 (ESXB) (Fig. 2B). SOMAmer ESXB 5557-2 was used to examine lysates from a time course study of human macrophages infected with M. tuberculosis strain H37Rv or M. bovis BCG, which lacks ESXB. Figure 2C clearly demonstrates that the ESXB aptamer signals increased with duration of infection for the H37Rv strain but not the ESXB-deficient BCG strain or uninfected control cells. ESXB was detectable as early as 6 h postinfection, where the bacterial load ranged from 1.2 × 10^4^ to 6.8 × 10^4^ CFU/ml, and the SOMAmer signals were just slightly yet significantly elevated (*P* = 0.0014) in lysates from H37Rv-infected compared to BCG-infected (control) macrophages. We also examined the ability of M. tuberculosis aptamers to detect their targets within culture filtrate proteins from three pathogenic M. tuberculosis strains. The observed median SBR values shown in Fig. 2D make it clear that the M. tuberculosis aptamers detected M. tuberculosis proteins within the CFPs of all three pathogenic strains tested in a concentration-dependent manner. Titrations of CFPs in serum indicated variable LODs for individual aptamers, ranging from 1.9 to 2,800 ng/ml CFP, corresponding to estimated bacterial loads of 3.4 × 10^5^ to 4.9 × 10^8^ cells/ml (Table 2). Taken together, the CFP and human macrophage lysate data clearly demonstrate that the M. tuberculosis pathogen SOMAmers detect natively expressed M. tuberculosis proteins.

### Examination of M. tuberculosis SOMAmer specificity.

**FIG 3:**
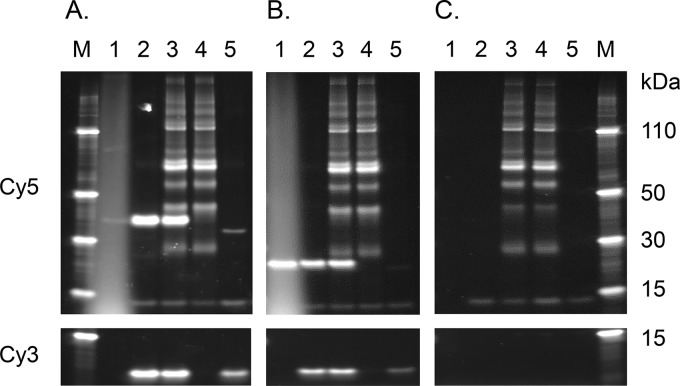
SDS-PAGE analysis of SOMAmer pulldown of recombinant and native M. tuberculosis proteins from buffer and 40% human serum. (A) A85A, 14504-6. (B) KAD, 14491-10. (C) Beads alone. Lane 1, recombinant target protein. Lanes 2 to 5, SOMAmer affinity pulldowns from buffer (lane 2), 40% human serum (lane 3), unspiked 40% human serum (lane 4), and native protein within HN878 CFPs (lane 5). (Upper) Alexa Fluor 647 dye-labeled proteins. (Lower) Cy3-labeled aptamers.

The specificity of the SOMAmer interactions was examined with pulldown experiments from buffer, human serum, or CFP, and recombinant forms of M. tuberculosis proteins were spiked into these matrices (Fig. 3). Bead-immobilized SOMAmers A85A (14504-6) and KAD (14491-10) successfully pulled down their recombinant targets spiked into buffer or serum (Fig. 3A and B, upper, lanes 2 and 3) and were also able to retrieve the native form of their targets from CFPs (Fig. 3A and B, upper, lane 5). In this rather crude pulldown assay, numerous serum proteins bound to control agarose beads in the absence of SOMAmers and appeared as nonspecific bands (Fig. 3C, upper, lanes 3 and 4).

Examination of the pattern of aptamer retention in pulldown provided an additional measure of target specificity. SOMAmer-target interactions are expected to occur with 1:1 stoichiometry based on crystallographic analyses that demonstrated interactions of SOMAmers with surface moieties on their target proteins (41). As can be seen in Fig. 3A and B, the target protein (upper) and the SOMAmer (lower) were retained in the pulldown eluates in roughly equivalent amounts, as predicted. Also notable is the absence of aptamer signal from the serum-alone lanes (lane 4), indicating that in the absence of its cognate target, the reagent was not significantly retained by nonspecific interaction with serum proteins.

To confirm that the M. tuberculosis aptamers were pulling down their intended targets, 8 top-performing reagents were utilized in pulldown experiments and eluates were analyzed by LC-MS/MS. Based on the observed peptides, both the native proteins from CFPs (20 μg) and the recombinant proteins (10 nM) were pulled down from buffer or 40% human serum (Table 3). One exception was CH602, where only the recombinant form was observed in the pulldown eluates, which indicates low abundance of the native form of this intracellular heat shock protein in CFPs.

**TABLE 3:** M. tuberculosis-derived peptides identified by LC-MS/MS in pulldown eluates from buffer or 40% human serum spiked with recombinant or native M. tuberculosis proteins

Target; SOMAmer used for pulldown	Protein identified by LC-MS/MS		No. of peptides observed in pulldowns of spiked serum			
			Recombinant protein (10 nM)		Native CFP (20 μg)	
	Name	Accession no.; locus designation	Buffer	Serum (40%)	Buffer	Serum (40%)
A85A; 14504-6	A85A	P9WQP3; A85A_MYCTU	33	29	145	191
A85B; 12074-5	A85B	P9WQP1; A85B_MYCTU	14	14	57	63
A85C; 14494-124	A85C	P9WQN9; A85C_MYCTU	82	63	70	80
CH602; 7592-57	CH602	P9WPE7; CH602_MYCTU	5	4	0	0
DNAK; 7606-49	DNAK	P9WMJ9; DNAK_MYCTU	102	81	57	49
ESXB; 5557-2	ESXB	P9WNK5; ESXB_MYCTU	25	19	10	7
MTB12; 15001-2	MTB12	P9WIN7; MTB12_MYCTU	6	0	12	5
RL7; 7596-2	RL7	P9WHE3; RL7_MYCTU	26	23	28	14

### Examination of the diagnostic utility of M. tuberculosis SOMAmers.

**FIG 4:**
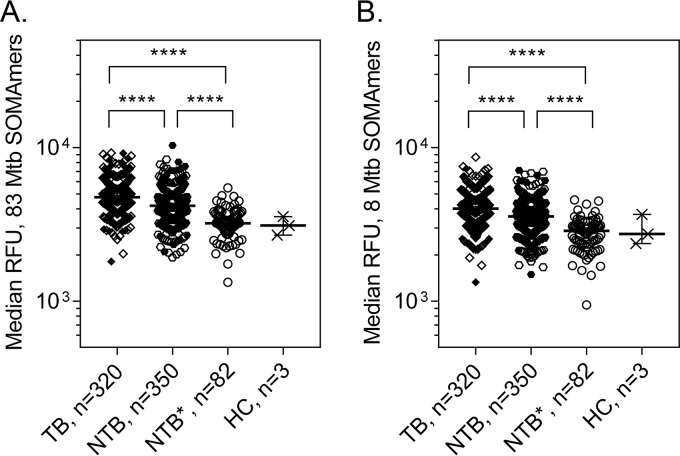
Median M. tuberculosis SOMAmer signals obtained via full SOMAscan assay. TB serum samples were compared to NTB, NTB* (non-TB samples from low-burden countries), and HC (healthy control) samples. RFU values were analyzed for all 83 M. tuberculosis samples (A) or were restricted to 8 M. tuberculosis aptamers described in Table 3 (B). Open symbols, HIV negative; solid symbols, HIV positive. ****, *P* < 0.0001. KS for TB versus NTB, 0.236 (A) and 0.233 (B).

Our next goal was to apply the M. tuberculosis aptamers in a diagnostic test for human pulmonary TB. We used the high-throughput SOMAscan and TB SOMApanel proteomic assay to examine differences between samples from culture-positive TB patients and from TB suspects that had subsequently been ruled out for TB based on culture and follow-up (NTB). A set of 740 serum samples was provided by the Foundation for Innovative New Diagnostics (FIND) and included samples from 354 TB and 386 NTB subjects of diverse geographic origins where the TB burden is moderate to very high (Bangladesh, Colombia, Peru, South Africa, Uganda, Vietnam, and Zimbabwe). About one-third (32.6%) of the samples were from HIV-positive subjects (Table S5A). The entire sample set was tested on full SOMAscan that incorporated the M. tuberculosis aptamers that had been characterized with regard to sensitivity and specificity as described above. After exclusion of hemolyzed samples, duplicates, and assay failures due to technical issues, SOMAscan data for 320 TB and 350 NTB samples were analyzed. Robust and reproducible differences in signals for host protein SOMAmers were observed between TB and NTB populations, as reported elsewhere (20). However, only minimal differential signals were observed for M. tuberculosis aptamers when examining median signals from 83 reagents (Fig. 4A) or from the 8 M. tuberculosis aptamers with liquid chromatography-tandem mass spectrometry (LC-MS/MS)-confirmed specificity as described in Table 3 (Fig. 4B). In both cases, comparisons of the medians indicated that they were significantly different (*P* < 0.0001), but given the overlap of the signal distributions (Kolmogorov-Smirnov statistic [KS] of <0.30), the separation between the median signal levels was too small to be of practical use. Control samples collected from patients with lung disorders other than TB from geographical areas (Spain and Canada) where TB burden is low (NTB*) and from healthy controls (HC) had significantly lower median signals than either TB or NTB study samples (*P* < 0.0001) (Fig. 4). When comparing TB with NTB* or HC, better separation of the signal distributions was observed in both the full set of 83 M. tuberculosis aptamers (Fig. 4A) (KS of 0.659) and the subset of 8 M. tuberculosis reagents (Fig. 4B) (KS of 0.603).

**FIG 5:**
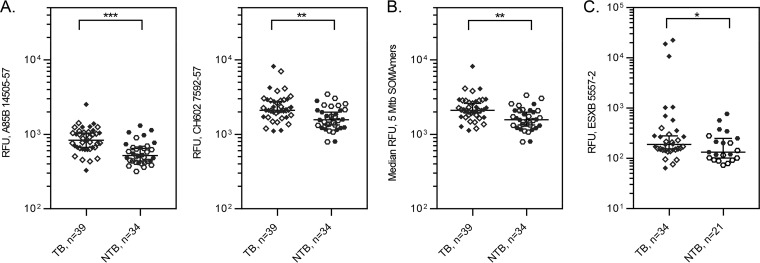
M. tuberculosis aptamer signals in TB versus NTB samples assayed on SOMApanels. (A) Signals from two individual M. tuberculosis SOMAmers in serum. **, *P* = 0.0063 and KS = 0.398; ***, *P* < 0.0003 and KS = 0.497. (B) Median of the top 5 M. tuberculosis aptamers on SOMApanel in serum. **, *P* < 0.0046 and KS = 0.4087. (C) ESXB SOMAmer signals observed in urine samples from TB and NTB samples. *, *P* < 0.0276 and KS = 0.4062. Open symbols, HIV negative; solid symbols, HIV positive.

To explore the differences between TB and NTB samples in greater detail, we designed a targeted TB SOMApanel which utilized a reduced set of 44 SOMAmers for M. tuberculosis proteins, 108 human proteins, and 75 controls. Serum samples from 39 TB and 34 NTB subjects collected in Peru, South Africa, and Vietnam, including samples from individuals coinfected with HIV, were tested on the TB SOMApanel (Table S5). When examining median signals across all 44 M. tuberculosis aptamers or the 8 M. tuberculosis reagents described in Table 3, the difference in the medians between TB and NTB did not reach statistical significance (*P* = 0.0906 and 0.2021, respectively). However, 5 SOMAmers targeting 5 M. tuberculosis proteins yielded statistically significantly elevated signals in TB samples, including A85B 14505-57 and CH602 7592-57 (*P* = 0.0003 and 0.0063, respectively) (Fig. 5A). When using the median of the signals from these 5 SOMAmers to compare the TB and NTB populations, statistical significance was observed in TB samples examined by SOMApanel (*P* = 0.0046; KS of 0.408) (Fig. 5B) and SOMAscan (data not shown).

Urine samples (*n* = 55) were examined on the TB SOMApanel. Only one M. tuberculosis SOMAmer, ESXB 5557-2, yielded differential signals in the urine concentrates between the TB and NTB groups, although with inadequate separation of the distributions (Fig. 5C).

**FIG 6:**
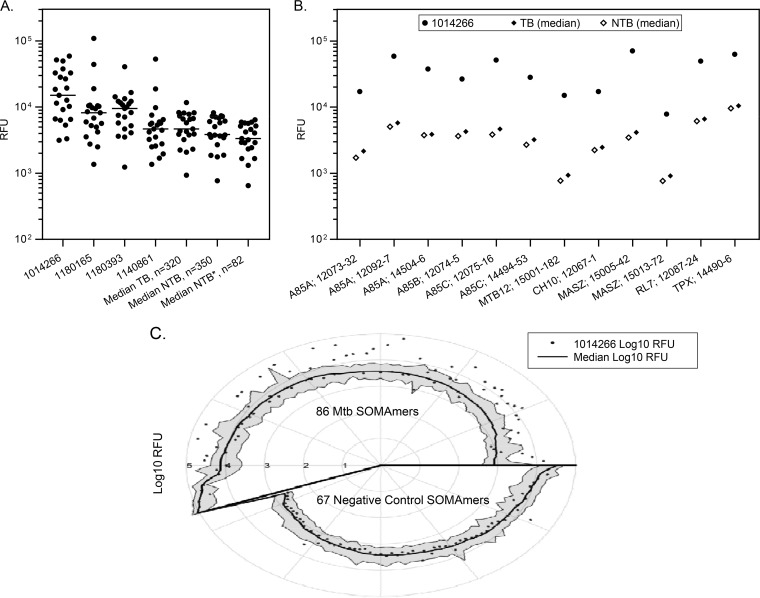
Unique serum samples that yielded high signals for numerous M. tuberculosis targets. (A) Signals for 21 M. tuberculosis aptamers in serum samples from four outlier patients compared to the median signals for TB, NTB, and NTB* (non-TB samples from low-TB-burden countries) patients. (B) Elevated signals for 12 M. tuberculosis aptamers in a serum sample from patient 1014266 compared to the median signal in TB (*n* = 320) and NTB (*n* = 350) samples. (C) Log_10_ RFU signal levels of all 86 M. tuberculosis SOMAmers in the upper region and negative controls (spuriomers and other nonhuman proteins) in the lower region for patient 1014266 are shown as dots. The median signal and the empirical 95% confidence intervals for all samples tested in this set (*n* = 403) are shown as black lines and the surrounding shaded region, respectively. The spiral pattern emerges from sorting the median signal levels from low to high.

Serum samples from four individuals yielded elevated signals for numerous M. tuberculosis antigens when examined by SOMAscan (Fig. 6A), and these data were confirmed by SOMApanel. Subject 1014266, a 60-year-old male from Peru with smear-negative pulmonary TB and HIV coinfection who was reported to be moderately ill, provided the most dramatic example, with many M. tuberculosis SOMAmer signals elevated in both serum (Fig. 6B) and plasma (data not shown). There were no indications that these samples were compromised or mishandled in either assay, and there were no discrepancies in the clinical and demographic metadata. These subjects were from South Africa, Vietnam, and Peru, and all were TB culture positive, coinfected with HIV, and mildly to moderately ill. Three of the four (except 1014266) were sputum smear positive. Furthermore, as exemplified by SOMAscan data for subject 1014266 from Peru, SOMAmers to nonhuman targets other than M. tuberculosis proteins, including negative-control probes (spuriomers), produced typical low signals (Fig. 6C). These data are suggestive of specific detection of elevated levels of M. tuberculosis proteins in these samples, although additional experiments are required to support this conclusion.

## DISCUSSION

Rapid and accurate TB diagnosis using pathogen markers remains a difficult goal. Detection of MPT64 and antigen 85B via immunostaining of tissues and fine-needle aspirates can be rapid and sensitive methods for establishing an early and specific diagnosis of TB infection but are limited to pathology and microbiology laboratories (42, 43). Detection of MPT64 in sputum cultures utilizing an immune-chromatographic assay, in conjunction with cording in a stained smear, has been used to make a preliminary yet high-confidence identification of M. tuberculosis complex in liquid culture (44, 45). Thus, localization of infection and the low bacterial burden in TB disease usually restricts the specimen type for direct detection methods to sputum or invasively acquired tissues. Direct antigen detection in blood or urine, however, has had very limited success for TB diagnosis in the past, and this shortcoming has been attributed, in part, to insufficient sensitivity of the reagents and assay platforms and to the lack of optimal sample processing (6). Lipoarabinomannan (LAM) diagnostic tests for urine specimens have been developed, although they tend to suffer from poor sensitivity except in TB patients coinfected with HIV (46). Using multiple reaction monitoring-mass spectrometry (MRM-MS), the Dobos group has identified numerous M. tuberculosis proteins in exosomes purified from human serum which are actively being pursued as diagnostic biomarkers of M. tuberculosis infection (23, 47). Campos-Neto and colleagues have identified several M. tuberculosis proteins in large urine volumes by mass spectrometry, most notably Rv1681, but to our knowledge these efforts have not moved into diagnostic development (48–50).

The goal of this study was to develop highly sensitive and specific SOMAmer reagents for use in a diagnostic assay capable of distinguishing active TB infection from latent TB infection (LTBI) and NTB when utilizing serum or urine specimens. For aptamer selection, we chose a defined set of 18 recombinant M. tuberculosis proteins instead of native CFPs for ease of tagging and consistent, reproducible production, although CFPs might have led to greater target diversity, including relevant M. tuberculosis-specific posttranslational modifications. Serum counterselection proved to be very effective for the development of highly specific M. tuberculosis aptamers by enabling removal of sequences with affinity binding to serum proteins. Binding studies with recombinant M. tuberculosis proteins spiked into buffer, serum, and urine protein concentrates established the sensitivities for the M. tuberculosis aptamers. As expected, LODs were lowest in buffer and ranged from 0.08 to 7.39 pM. Picomolar LODs were also obtained in the highly complex matrix of 40% serum (0.07 to 5.38 pM). In 40% serum, SOMAmer 14494-124 had the lowest LOD, 0.07 pM, or ∼5 pg/ml of antigen 85C in serum, which is roughly 3 orders of magnitude below reported LOD values for indirect enzyme-linked immunosorbent assay (ELISA) using anti-antigen 85 antibodies (51, 52) but about 3 orders of magnitude above the LOD of antigen 85B detection by immuno-PCR (53). LODs in urine protein concentrates ranged from 0.06 to 55.47 pM. With the exception of KAD and A85B, most M. tuberculosis aptamers showed urine LODs consistent with those obtained in 40% serum. As was the case with serum, it is very likely that SELEX counterselection using urine protein concentrates would allow for the development of KAD and A85B SOMAmers with greater sensitivities in urine.

M. tuberculosis aptamers also identified several natively generated pathogen proteins within lysates of M. tuberculosis-infected human macrophages and in M. tuberculosis culture filtrates, which demonstrated that the M. tuberculosis aptamers recognize the native forms of the proteins. Our results are consistent with a published study on the proteomic analysis to identify highly antigenic proteins on exosomes from M. tuberculosis-infected macrophages (23). Differences were observed in the M. tuberculosis SOMAmer signal intensities when using CFP fractions from three pathogenic M. tuberculosis strains, but it is unclear whether these differences reflect variable relative abundance of the target proteins or are due to variable aptamer affinities for the targets from different strains.

SOMAmers recognize structural features on the surface of their target proteins (41, 54). These epitopes may be masked during an infection due to interactions with antibodies and other proteins. We evaluated methods for immune complex dissociation that retain the integrity of the target epitopes (mild acid, increased temperature, and detergents) as well as different assay and buffer conditions (see Table S6 in the supplemental material). These efforts were largely unsuccessful toward overall improved SOMAmer-based detection of M. tuberculosis proteins. Related to that, we considered using depletion columns or affinity enrichment to augment detectability of low-abundance pathogen protein detection, but such methods would add extra steps and time, which is incompatible with the development of a patient-near test.

Several M. tuberculosis SOMAmers had elevated background levels compared to typical low signals for negative-control aptamers (spuriomers). Given the conservation of structural motifs in widely divergent proteins, we sought to examine potential cross-reactivity of the M. tuberculosis SOMAmers with human proteins in serum via pulldown assays. Like SOMAscan, pulldowns employ two separate catches of the formed target-SOMAmer complexes and thus achieve high specificity through removal of nonspecifically bound proteins (catch 1) and then further cleanup of the bound reagents (catch 2) before readout of the signals. On SOMAscan, SOMAmer hybridization to its complementary probe on an array provides the signal. In pulldown, bead-bound protein-SOMAmer complexes were directly assayed via SDS-PAGE and thus show background proteins present in catch 1-only fractions. Additionally, the pulldown assays also utilized individual, more concentrated (10 nM versus 0.5 nM) SOMAmers on agarose beads instead of the 200 or 4,000-plus reagents in the M. tuberculosis SOMApanels or SOMAscan, respectively. Still, the pulldown experiments using samples spiked with 10 nM protein demonstrated that the SOMAmers are highly selective affinity reagents. For a small subset of clinical samples, pulldown followed by LC-MS/MS formats were attempted, although we were unable to confirm the presence of M. tuberculosis proteins in these samples by these methods, likely due to the less sensitive method of MS (data not shown).

Having established the sensitivity and specificity of the M. tuberculosis aptamers, we utilized them to interrogate well-characterized TB and NTB samples. Many samples from TB patients had elevated signals for one or several M. tuberculosis aptamers, but the identity and levels of the antigens were highly variable and reminiscent of observations limiting the use of serology in TB diagnosis. Moreover, it appeared that the M. tuberculosis aptamer signals did not separate confirmed TB from TB suspects that were ruled out for TB (NTB) but were, nonetheless, clearly different from control patients from low-TB-burden areas (NTB*) or from healthy controls (HC). It is possible that M. tuberculosis bacilli present in the granulomas in the lungs of patients with chronic TB release very little M. tuberculosis protein into circulation and that a substantial fraction of these proteins is rapidly modified, complexed, or degraded (55–57) and thus is no longer detectable by SOMAmers in serum or urine. Furthermore, the M. tuberculosis SOMAmers were raised against recombinant targets, and while we have demonstrated that the M. tuberculosis SOMAmers recognize native M. tuberculosis proteins generated in culture and within infected macrophages, it remains possible that differences in protein folding and posttranslational modifications reduce aptamer binding efficiencies to native M. tuberculosis proteins in serum or urine. Given that 30% of the world population and up to 80% of the population in some regions where TB is endemic are latently infected with M. tuberculosis (1), one possible explanation for the elevated NTB signals is that many of the non-TB patients in this study had LTBI or nontuberculous mycobacterial infection and had some level of circulating mycobacterial proteins in their serum and urine. Our estimate for bacterial load resulting in detection of M. tuberculosis proteins by SOMAscan is in the range of 10^4^ to 10^5^ bacilli/ml, based on fractions from macrophage infection models and CFP preparations. We found very few serum samples which produced highly elevated signals of the M. tuberculosis SOMAmers. In one patient from Peru with culture-positive, smear-negative TB and HIV coinfection, dozens of aptamer signals were 4- to 10-fold above the median signal observed in TB samples. These observations were confirmed in separately acquired aliquots of both serum and plasma, although the M. tuberculosis proteins were not observed in affinity capture eluates examined by LC-MS/MS. Based on standard curves using recombinant proteins run in the same experiments, even with greatly elevated signals well above the LODs for M. tuberculosis SOMAmers, the estimated antigen levels in this sample were only in the low picomolar range. Thus, confirmation of the signals by MS may require a more advanced approach, such as targeted MRM-MS. It is not known whether this patient had an M. tuberculosis bloodstream infection that could explain such high levels of circulating antigens (58). In support of this hypothesis, peptides corresponding to the M. tuberculosis proteins antigen 85B, antigen 85C, Apa, BfrB, GlcB, HspX, KatG, and Mpt64, several of which were on our list of targets, had been previously identified by targeted MRM-MS assays of exosomes from TB patients (47, 59). That study also found a high variability in the identity of the corresponding targets and in the number of peptides detected, and some of the peptides (e.g., from MPT64) were also present in exosomes from LTBI. An additional possible explanation for the elevation of SOMAmer signals in NTB samples compared to signals in control sample groups is aptamer cross-reactivity with homologous proteins from NTM or from other actinobacteria.

In conclusion, a collection of M. tuberculosis aptamer reagents with picomolar affinities were generated. However, the differences in the M. tuberculosis aptamer signals between TB and NTB in serum or urine were too small to be diagnostically useful. Some of the M. tuberculosis SOMAmer reagents are currently being evaluated, along with aptamers for host TB markers, on other sensitive assay platforms, including sandwich-based assays. Since M. tuberculosis is an intracellular pathogen, samples such as whole blood, buffy coat, or dried blood spots may be better matrices to detect pathogen-derived products. In contrast, strong and robust differences in the expression of human host response proteins were observed between TB and TB-suspect populations by SOMAscan and SOMApanel, as reported in the accompanying paper by De Groote et al. (20).

## MATERIALS AND METHODS

### Buffers and reagents.

Serum sample diluent (SSD) is composed of 40 mM HEPES, 61 mM NaCl, 3 mM KCl, 5 mM MgCl_2_, 0.6 mM EGTA, 1.2 mM benzamidine, 0.02 mM Z-block (see below), and 0.05% (vol/vol) Tween 20, adjusted to pH 7.5 with NaOH. Buffer SB18T is composed of 40 mM HEPES, 120 mM NaCl, 5 mM KCl, 5 mM MgCl_2_, and 0.05% (vol/vol) Tween 20, adjusted to pH 7.5 with NaOH. Buffer SB17T is SB18T supplemented with 1 mM EDTA.

Z-block is a SOMAmer mimic, was used at 0.1 to 20 μM to block nonspecific interactions of reagents with proteins, and was prepared at SomaLogic. Dextran sulfate (Sigma) was another polyanionic competitor and was prepared as a 10 mM solution in SB17T.

### M. tuberculosis proteins and culture filtrate proteins.

Eight M. tuberculosis genes were PCR amplified from genomic DNA of strain H37Rv (NR-14865; BEI Resources) using KOD XL DNA polymerase (EMD Millipore) and ligated into pET-51b vector (EMD Millipore) using restriction sites contained in the primer sequences (see Table S1 in the supplemental material). For 10 additional targets, pET-15b- or pET-23a-based expression vectors were available from BEI Resources (Table S1). All plasmids were sequenced (SeqWright) to verify the identity of the cloned genes and their proper in-frame fusion with the vector-encoded tag(s). Escherichia coli Rosetta (EMD Millipore) was transformed with these plasmids and grown in 400 ml of LB medium for the production of bacterial proteins as previously described (60, 61). In brief, expression was induced with 0.5 mM isopropyl-β-d-thiogalactopyranoside (IPTG) during mid-exponential growth (optical density at 600 nm of 0.3 to 0.7), and cultures were shaken for 4 to 16 h at 30 to 35°C. After cell lysis with BugBuster/Benzonase reagent (EMD Millipore), the recombinant His-tagged proteins were purified from the soluble fraction via affinity chromatography on nickel-nitrilotriacetic acid (Ni-NTA) agarose. Proteins that also contained a Strep tag were further purified using StrepTactin Superflow agarose (EMD Millipore). All 18 M. tuberculosis proteins were obtained in sufficient amounts and purity for use in SELEX and subsequent binding and spiking assays (Table S1).

Culture filtrate proteins (CFP) from M. tuberculosis strains H37Rv, CDC1551, and HN878 were obtained from BEI Resources (Manassas, VA), and their preparation was described previously (62). The strains represent a laboratory-adapted isolate (H37Rv) and a clinical isolate (CDC1551) from the Euro-American lineage and a clinical isolate (HN878) from the East Asian lineage (63). Recovery of CFP for these strains by this method ranged from 11 to 12 μg/mg of wet cell paste, with roughly 8 × 10^8^ cells/mg of wet cell paste.

### SOMAmer selection and synthesis.

Seven different modified nucleotide libraries were used for SELEX, including 2NapdU, 2NEdU, BndU, NapdU, PEdU, PPdU, and TrpdU (64), using methods previously described (19, 60). Special effort was devoted to generating M. tuberculosis SOMAmer reagents with minimal nonspecific background in serum by applying counterselection with serum competitor buffer (SCB; 1 μM casein, 1 μM prothrombin, 7.5% serum) and with protein competitor buffer (PCB; 1 μM casein, 1 μM prothrombin, 1.5 μM albumin) in alternating SELEX rounds. For some selections of antigen 85 (A85) SOMAmers, complexes with fibronectin (FN) were preformed by mixing the two proteins together in 1:1 stoichiometric ratios and including a 4-fold molar excess of untagged, free fibronectin for counterselection during SELEX. For all targets, increasingly longer (up to 90 min) kinetic challenges with 10 mM dextran sulfate were performed to favor slow off-rates. SOMAmer-target complexes were partitioned with paramagnetic Dynabeads His tag beads (ThermoFisher) that bind the His tag on the recombinant proteins, and selected aptamers were amplified using KOD XL DNA polymerase. Aptamer pools with good affinity (equilibrium dissociation constant [*K_D_*] of ≤10 nM) were deep sequenced, and enriched sequences were prepared synthetically as 50-mers via standard phosphoramidite chemistry, incorporating 5′PBDC (photocleavable biotin, D spacer, cyanine 3) and an inverted dT nucleotide at the 3′ end (3′idT) for added stability to 3′ to 5′ exonucleases. After initial characterization, the best SOMAmers were prepared at larger scale (1 μmol) and purified by high-performance liquid chromatography (HPLC).

### SOMAmer characterization.

SOMAmers were refolded by denaturation for 5 min at 95°C, followed by cooling to room temperature over a 10- to 15-min period. For equilibrium solution binding assays, ^32^P-radiolabeled aptamers (10 to 50 pM) were incubated for 2 h at 37°C with serially diluted proteins (0.001 to 100 nM). Zorbax PSM-300A (Agilent Technologies) resin or Dynabeads His tag beads (ThermoFisher) were used for partitioning of the complexes. Aptamer affinities were calculated from the resulting binding curves and expressed as *K_D_*s, along with the maximum bound fraction (binding plateau) that is influenced by the retention efficiency of the target-SOMAmer complexes on Zorbax, the fraction of binding-competent aptamers, and traces of unincorporated radiolabel. Apparent *K_D_* (*K_D_*, app) values were determined in the proteomic SOMAscan assay (described below), where each aptamer was present at higher concentration (500 pM) and the equilibration time was longer (3.5 h).

### Affinity capture and LC-MS/MS protein identification.

Affinity capture assays (pulldowns) were performed as previously reported (19), with modifications. Agarose-streptavidin beads, with 10 pmol of preimmobilized SOMAmers, were preblocked (SB17; 1% bovine serum albumin [BSA], 2 mg/ml sheared salmon sperm DNA) for 1 h at 850 rpm and room temperature. After washing, unoccupied streptavidin was blocked with biotin (SB17T; 10 mM biotin). Recombinant proteins (25 nM) and CFPs (20 μg) were diluted in serum (40% final concentration) or buffer and SSD (1× final concentration). Subject serum samples were likewise diluted to 40% in 1× SSD. Samples were added to the SOMAmer beads and incubated for 3 h at 850 rpm and 37°C. Unbound or nonspecifically retained proteins were removed by washing with 10 mM dextran sulfate and three washes with SB17T (5 min, 850 rpm). Captured targets were labeled with 200 μM EZ Link NHS-PEO4-biotin (Pierce) and 50 μM NHS-Alexa Fluor 647 dye (Invitrogen) for 5 min. After quenching and washing, complexes were released from the beads via photocleavage of the biotin-SOMAmer linkage (UV exposure, 20 min, 850 rpm) and then captured on Dynabeads MyOne streptavidin beads (Invitrogen). Traces of nonbiotinylated protein, nonspecifically retained reagents, and free SOMAmers were removed by sequential washing with 30% glycerol (in SB17T) and SB17T. Protein-SOMAmer complexes were eluted from the beads by boiling in 1× SDS-PAGE sample buffer (Invitrogen) and analyzed by SDS-PAGE, and fluorescence was imaged using cyanine-3 (aptamer reagents) and cyanine-5 (Alexa Fluor 647 dye-labeled protein) settings.

Affinity capture eluates were also evaluated by LC-MS/MS, and tandem mass spectra were collected and processed at MSBioworks (Ann Arbor, MI). For detergent removal, eluates were loaded onto a 10% Bis-Tris SDS-PAGE gel (Novex, Invitrogen) and separated by approximately 1 cm. The gel was stained with Coomassie, the entire mobility region was excised into one segment, and in-gel trypsin digestion was performed. Digests were analyzed by nano-LC-MS/MS with a Waters NanoAcquity HPLC system interfaced to a ThermoFisher Q Exactive. Peptides were loaded on a trapping column and eluted over a 75-μm analytical column at 350 nl/min; both columns were packed with Jupiter Luna C_18_ resin (Phenomenex). A 1-h gradient was employed. The mass spectrometer was operated in data-dependent mode, with MS and MS/MS performed in the Orbitrap at 70,000-FWHM (full width at half maximum) resolution and 17,500-FWHM resolution, respectively. The 15 most abundant ions were selected for MS/MS. Data were searched using a local copy of Mascot with the following parameters: enzyme, trypsin; database, Swiss-Prot human and MYCTU sequences (forward and reverse appended with common contaminants); fixed modification, carbamidomethyl (C); variable modifications, oxidation (M), acetyl (protein N-term), deamidation (NQ), and Pyro-Glu (N-term Q). The following mass values were used: monoisotopic peptide mass tolerance, 10 ppm; fragment mass tolerance, 0.02 Da; maximum missed cleavages, 2. Mascot DAT files were parsed into the Scaffold software for validation and filtering and to create a nonredundant list for each sample. Data were filtered with 1% protein and peptide false discovery rates and required at least two unique peptides per protein.

### SOMAscan and SOMApanel arrays for proteomic analysis.

Proteomic analysis was performed as previously described (19, 65), except where assay conditions were modified or samples underwent pretreatment, as indicated. Briefly, serum samples (50 μl) were mixed with specific SOMAmer binding reagents to form complexes and subjected to a two-capture assay, followed by elution and quantitation of the bound reagents via hybridization to microarrays. Automated SOMAscan V3 (SomaLogic, Inc.) was applied for this study to measure 4,000 human proteins (R&D Plex) and 18 M. tuberculosis pathogen products, using high-density hybridization microarrays from Agilent Technologies, Santa Clara, CA. Additionally, focused TB SOMApanel slides were manufactured by Applied Microarrays, Tempe, AZ. These low-density microarrays contained complementary probes for 86 M. tuberculosis aptamers in version 1, which were further pared down to 43 probes in version 2. Quality control measures included control aptamers for data normalization, hybridization controls to measure hybridization efficiency, and calibration samples to control for assay variability.

Signal-to-background ratios (SBRs) were calculated from titrations of purified recombinant M. tuberculosis protein and native M. tuberculosis CFPs spiked in matrix (serum, urine, and buffer) as the ratio of the net RFU to the background RFU signals as (RFU_spike_ − RFU_background_)/RFU_background_.

The limit of detection (LOD) and limit of quantitation (LOQ) were determined using the limit-of-the-blank method, where LOD is the mean blank plus 3.3 times the standard deviation blank (one-sided 95% confidence interval times 2) and LOQ is the mean blank plus 10 times the standard deviation blank (one-sided 95% confidence interval times 6) (66). LOD and LOQ studies utilized 16-point titration curves of M. tuberculosis recombinant proteins spiked into matrix (buffer, 40% pooled human serum, or 100 μg urine protein concentrates from urine samples pooled from participants with TB and TB suspects that had been ruled out for TB [NTB] based on culture and follow-up).

### Sample preparation.

Serum and urine samples were provided by the Foundation for Innovative New Diagnostics (FIND; Geneva, Switzerland). Serum samples were thawed and stored on ice, and aliquots (50 μl) were diluted to 40% in SSD (described above). Several methods for immune complex dissociation were evaluated (67, 68), including pretreatment of samples with mild acid (HCl or glycine to pH 3 for 10 to 30 min, followed by neutralization with NaOH to pH 8), elevated temperature (56°C, 30 min), or with detergents such as Tween 20, Triton X-100, and CHAPS {3-[(3-cholamidopropyl)-dimethylammonio]-1-propanesulfonate} (0.1 to 1%).

Urine samples (2 ml) were thawed and treated with 1 M Tris-Cl, pH 8 (80 mM final concentration), to solubilize cryoprecipitates. Samples were concentrated and buffer exchanged twice into SB17T by ultrafiltration (3,000 molecular weight cutoff; Millipore), resulting in an approximately 100-μl final volume. Protein concentration was determined by the Bradford method (Quick Start Bradford; Bio-Rad), and 50-μg urine protein concentrates were examined by SOMApanel.

Fractions from a macrophage model of *in vitro* M. tuberculosis infection were prepared as follows. Human macrophages (THP-1) were infected with M. tuberculosis (multiplicity of infection of 5:1 or 10:1) for 4 h, and then nonphagocytized bacteria were removed, the infected cells were washed, and fresh medium was added. Cells were harvested at 6, 24, and 72 h postinfection, and lysates (1 ml) were prepared with a lysis buffer containing 0.05% SDS, passed through a 0.2 μm filter, dialyzed against 10 mM ammonium bicarbonate, and immediately stored at −80°C. SDS removal spin columns (SDS-Out; Pierce) were used to remove SDS from the lysates, and 1.3 μg of lysates was used in the TB SOMApanel. Spent macrophage culture medium was also collected at 6, 24, and 72 h postinfection, filter sterilized, and frozen until proteomic analysis.

### Statistical analysis.

Statistical analysis was performed using tools available in GraphPad Prism 7. For multiple-group comparisons, analysis of variance with Tukey's correction for multiple comparisons was used. Multiplicity-adjusted *P* values of 0.05 (95% confidence interval) were considered significant. For two-group comparisons (TB and NTB), *t* tests (two-tailed, unequal variance) and the Kolmogorov-Smirnov (KS) tests were used to determine statistical significance of differences in the signal distributions. A *P* value of <0.05, without correction for multiple comparisons, was considered significant.

## Supplementary Material

Supplemental material
