# Supplementary material for: Potential of High-Affinity, Slow Off-Rate Modified Aptamer Reagents for Mycobacterium tuberculosis Proteins as Tools for Infection Models and Diagnostic Applications
Source: J Clin Microbiol. 2017 Sep 25;55(10):3072–88. doi: 10.1128/JCM.00469-17 (PMC5625393; doi:10.1128/JCM.00469-17)
Supplement: Supplemental material [file supp_55_10_3072__index.html]

Supplemental material 

# Potential of High-Affinity, Slow Off-Rate Modified Aptamer Reagents for Mycobacterium tuberculosis Proteins as Tools for Infection Models and Diagnostic Applications

## Supplemental material

- Supplemental file 1 -

  Table S1

  PDF, 128K
- Supplemental file 2 -

  Table S2 (SOMAmer reagents generated via SELEX with purified recombinant *M. tuberculosis* proteins)

  PDF, 56K
- Supplemental file 3 -

  Table S3 (Characterization of SOMAmer reagents generated via SELEX with recombinant antigen 85 proteins)

  PDF, 26K
- Supplemental file 4 -

  Table S4 (Effect of counterselection with human serum on affinity and background of *M. tuberculosis* SOMAmer reagents)

  PDF, 21K
- Supplemental file 5 -

  Table S5 (Demographic and clinical characteristics of the participants in this study)

  PDF, 48K
- Supplemental file 6 -

  Table S6 (SOMAscan assay variations and sample pretreatment methods applied for optimization of signal-to-background ratio)

  PDF, 20K
