# Supplementary material for: Potential of High-Affinity, Slow Off-Rate Modified Aptamer Reagents for Mycobacterium tuberculosis Proteins as Tools for Infection Models and Diagnostic Applications
Source: J Clin Microbiol. 2017 Sep 25;55(10):3072–88. doi: 10.1128/JCM.00469-17 (PMC5625393; doi:10.1128/JCM.00469-17)

TABLE S1 Cloning, over-expression and protein purification. Eight genes of *M. tuberculosis* H37Rv were PCR-amplified using the indicated primers and cloned as *Bam*HI-*Sac*I or *Hin*dIII-*Sac*I fragments into pET-51 (restriction sites present in the primers are shown in underlined italics). Expression plasmids for 10 other proteins were obtained from BEI Resources. The proteins were over-expressed in *E. coli* Rosetta and purified at the 1 mg scale via affinity chromatography using the His-tag and/or Strep-tag. Purity of the recombinant proteins was assessed by SDS-PAGE.

#	Gene	Protein	Accession No.	Size, recombinant (kDa)	Protein domain (amino acids)	Tag, amino-terminal	Tag, carboxy-terminal	Plasmid for overexpression	Plasmid Source
1	Rv3804c	A85A_MYCTU	P9WQP3	35.7	47-336	Strep	His10	pET-51b-T3804-fbpA	SomaLogic[1]
2	Rv1886c	A85B_MYCTU	P9WQP1	35.3	46-323	Strep	His10	pET-51b-T1886-fbpB	SomaLogic[2]
3	Rv0129c	A85C_MYCTU	P9WQN9	35.7	52-328	Strep	His10	pET-51b-T0129-fbpC	SomaLogic[3]
4	Rv2031c	ACR_MYCTU	P9WMK1	16.0	n/a	His6	none	pMRLB.15/ Rv2031c/HspX	BEI (NR-13274)
5	Rv0577	CF30_MYCTU	P9WIR3	27.0	n/a	none	His6	pMRLB.24/Rv0577	BEI (NR-13288)
6	Rv3418c	CH10_MYCTU	P9WPE5	10.0	n/a	none	His6	pMRLB.9/Rv3418c/GroES	BEI (NR-13282)
7	Rv0440	CH602_MYCTU	P9WPE7	65.0	n/a	none	His6	pMRLB.1/Rv0440/GroEL2	BEI (NR-13276)
8	Rv0350	DNAK_MYCTU	P9WMJ9	70.0	n/a	none	His6	pMRLB.6/Rv0350/DnaK	BEI (NR-13279)
9	Rv3875	ESXA_MYCTU	P9WNK7	15.2	1-95	Strep	His10	pET-51b-T3875-Esat6	SomaLogic[4]
10	Rv3874	ESXB_MYCTU	P9WNK5	15.8	2-99	Strep	His10	pET-51b-T3874-Cfp10	SomaLogic[5]
11	Rv0733	KAD_MYCTU	P9WKF5	29.0	n/a	none	His6	pMRLB.25/Rv0733/Adk	BEI (NR-13289)
12	Rv1837c	MASZ_MYCTU	P9WK17	80.0	n/a	none	His6	pMRLB.8/Rv1837c/GlcB	BEI (NR-13281)
13	Rv1980c	MP64_MYCTU	P9WIN9	25.0	n/a	His6	none	pMRLB.12/Rv1980c/Mpt64	BEI (NR-13273)
14	Rv3803c	MPT51_MYCTU	P9WQN7	33.0	35-298	Strep	His10	pET-51b-T3803-mpt51	SomaLogic[6]
15	Rv2376c	MTB12_MYCTU	P9WIN7	17.0	51-166	Strep	His10	pET-51b-T2376-cfp2	SomaLogic[7]
16	Rv0934	PSTS1_MYCTU	P15712	41.1	25-373	Strep	His10	pET-51b-T0934-pstS1	SomaLogic[8]
17	Rv0652	RL7_MYCTU	P9WHE3	13.0	n/a	none	His6	pMRLB.13/Rv0652/Rp1L	BEI (NR-13285)
18	Rv1932	TPX_MYCTU	P9WG35	17.0	n/a	none	His6	pMRLB.14/Rv1932/Tpx	BEI (NR-13286)

[1]Primers 5'-GCGC*GGATCC*GGGCTTGCCGGTGGAG, 3'-GCGC*GAGCTC*CCTGGGGCGCGG, PCR product 889 bp

[2]Primers 5'-GCGC*AAGCTT*CTGCCGGTCGAGTACCTGC, 3'-GCGC*GAGCTC*CTAACGAACTCTGCAGGTCAC, PCR product 852 bp

[3]Primers 5'-GCGC*AAGCTT*CCAGTGGAATATCTGCAGGTG, 3'-GCGC*GAGCTC*CGTTGAGCACATGCTGGATATC, PCR product 846 bp

[4]Primers 5'-GCGC*AAGCTT*ATGACAGAGCAGCAGTGGAATTTC, 3'-GCGC*GAGCTC*CGAACATCCCAGTGACGTTGC, PCR product 303 bp

[5]Primers 5'-GCGC*AAGCTT*GCAGAGATGAAGACCGATGCC, 3'-GCGC*GAGCTC*CCATTTGCGAGGACAGCGC, PCR product 312 bp

[6]Primers 5'-GCGC*AAGCTT*CCATACGAGAACCTGATGGTG, 3'-GCGC*GAGCTC*GGATCGCACCGACGATATC, PCR product 813 bp

[7]Primers 5'-GCGC*AAGCTT*GACCCGGCATCCGCCCCTGAC, 3'-GCGC*GAGCTC*TCCCTGCGGCCTGCAGCAACTC, PCR product 378 bp

[8]Primers 5'-GCGC*AAGCTT*GGCTCGAAACCACCGAGCG, 3'-GCGC*GAGCTC*TGGAAATCGTCGCGATCAACG, PCR product 1068 bp

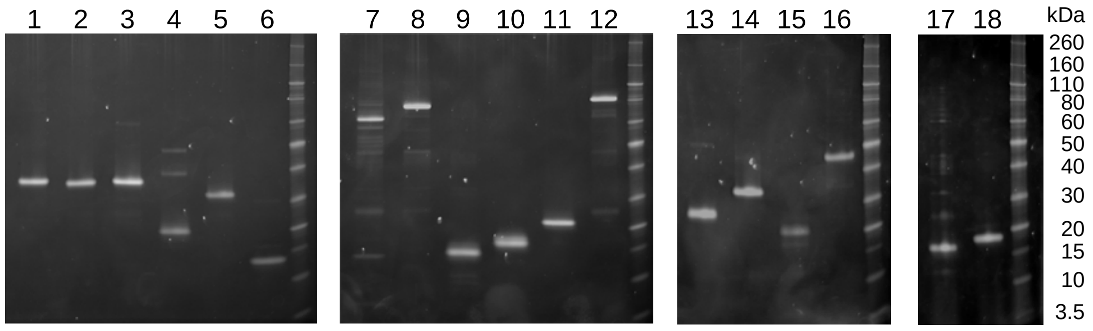

Supplement: Supplemental material [file JCM.00469-17_zjm999095670s1.pdf]
