# Supplementary material for: Potential of High-Affinity, Slow Off-Rate Modified Aptamer Reagents for Mycobacterium tuberculosis Proteins as Tools for Infection Models and Diagnostic Applications
Source: J Clin Microbiol. 2017 Sep 25;55(10):3072–88. doi: 10.1128/JCM.00469-17 (PMC5625393; doi:10.1128/JCM.00469-17)
Supplement: Supplemental material [file JCM.00469-17_zjm999095670s2.pdf]

TABLE S2 SOMAmer reagents generated via SELEX with purified recombinant Mtb proteins. Different modified nucleotide libraries were used in parallel, including BndU, NapdU, 2NapdU, 2NEdU, PEdU, PPdU, and TrpdU, resulting in multiple high-affinity SOMAmers for most targets. Affinity was determined by equilibrium binding assays (K_d) and also by SOMAscan assay ($K_{d,\ app}$).

Target (Gene)	Function or Name(s)	MW (kDa), native	SOMAmer[1]	Modified nucleotide	K_d (nM)	K_d app (nM)
A85A (Rv3804c)	Mycolyltransferase, antigen 85A, FbpA	31.7	4948-1	NapdU	0.05	0.005
			4953-64	TrpdU	0.06	0.018
			12073-8	NapdU	0.09	0.023
			12073-32	NapdU	0.16	0.012
			12092-7	TrpdU	0.06	0.007
			14492-7	2NapdU	0.24	0.045
			14492-11	2NapdU	0.21	0.160
			14504-6	PPdU	0.07	0.003
A85B (Rv1886c)	Mycolyltransferase, antigen 85B, FbpB	30.7	4949-52	NapdU	6.39	0.018
			4954-5	TrpdU	0.31	0.002
			12074-5	NapdU	0.08	0.002
			12074-11	NapdU	0.14	0.002
			12093-26	TrpdU	0.26	0.012
			14493-5	2NapdU	1.94	0.136
			14493-16	2NapdU	0.29	0.061
			14505-57	PPdU	0.13	0.032
A85C (Rv0129c)	Mycolyltransferase, antigen 85C, FbpC	32.1	4950-27	NapdU	0.03	0.002
			4955-49	TrpdU	0.26	0.080
			5569-2	2NapdU	0.01	0.005
			5575-1	PEdU	0.03	0.014
			12075-16	NapdU	0.05	0.019
			12075-40	NapdU	0.13	0.063
			14494-53	2NapdU	0.03	0.002
			14494-124	2NapdU	0.02	0.001
			14506-48	PPdU	0.04	0.017
			14506-76	PPdU	0.03	0.004
ACR (Rv2031c)	α-Crystallin, heat-shock protein HspX	16.1	7604-59	TrpdU	17.5	1.440
			7616-43	2NapdU	20.3	0.887
			14483-8	2NapdU	32.0	2.580
CF30 (Rv0577)	Culture filtrate protein 27	27.3	7610-49	TrpdU	5.41	0.334
			7618-50	2NapdU	2.20	0.531
			12089-13	TrpdU	1.83	0.019
CH10 (Rv3418c)	10 kDa chaperonin, GroES	10.8	7595-51	2NapdU	0.43	0.004
			7600-67	BndU	9.45	0.378
			7608-61	TrpdU	11.1	0.381
			12067-1	NapdU	7.55	0.439
			14488-1	2NapdU	0.53	0.005
			14488-3	2NapdU	0.05	0.003
CH602 (Rv0440)	60 kDa chaperonin GroEL2	56.7	7592-57	2NapdU	0.46	0.017
			7605-11	TrpdU	1.84	0.236
			14484-4	2NapdU	0.50	0.010
			14484-33	2NapdU	3.03	0.179
			14498-14	PPdU	7.39	0.388

TABLE S2 (continued)

Target (Gene)	Function or Name(s)	Size (kDa), native	SOMAmer[1]	Modified nucleotide	K_d (nM)	K_d app (nM)
DNAK (Rv0350)	Chaperone, Hsp70	66.8	7606-49	TrpdU	0.66	0.039
			14486-2	2NapdU	1.03	0.007
ESXA (Rv3875)	ESAT-6, early secretory antigen 6	9.8	7612-56	TrpdU	41.4	1.580
			7620-5	2NapdU	54.7	0.791
ESXB (Rv3874)	ESAT-6-like protein EsxB, CFP10	10.8	5557-2	2NapdU	0.54	0.004
			5562-95	PEdU	4.52	0.791
KAD (Rv0733)	Adenylate kinase	20.1	7598-15	2NapdU	1.11	0.051
			12090-3	TrpdU	13.2	0.376
			14491-10	2NapdU	0.08	0.001
			14491-43	2NapdU	0.12	0.001
			14502-11	PPdU	1.28	0.895
			14502-14	PPdU	0.83	0.319
MASZ (Rv1837c)	Malate synthase, GlcB	80.4	14487-46	2NapdU	6.60	1.180
			14499-2	PPdU	8.84	0.574
			14999-10	NapdU	5.27	0.592
			14999-49	NapdU	4.67	1.990
			15005-35	TrpdU	4.61	2.070
			15005-42	TrpdU	4.08	1.590
			15013-1	BndU	8.06	2.930
			15013-72	BndU	8.54	0.672
MP64 (Rv1980c)	Secreted immunogenic protein Mpt64	22.4	7615-18	2NapdU	12.3	1.440
			14496-43	PPdU	2.07	0.028
MPT51 (Rv3803c)	Fibronectin-binding protein Mpt51	28.5	5560-59	2NapdU	13.9	0.764
			7619-25	2NapdU	11.1	0.689
			14503-5	PPdU	21.1	1.410
MTB12 (Rv2376c)	Low molecular weight antigen, CFP-2	16.6	15001-2	NapdU	0.04	0.003
			15001-29	NapdU	0.06	0.004
			15001-182	NapdU	0.05	0.004
			15007-1	TrpdU	0.24	0.010
			15007-43	TrpdU	0.18	0.008
			15007-48	TrpdU	0.18	0.002
PSTS1 (Rv0934)	Phosphate-binding protein	35.9	5558-86	2NapdU	11.5	0.243
			7622-15	2NapdU	6.68	0.012
			14485-44	2NapdU	5.75	5.080
			14485-59	2NapdU	9.10	0.533
RL7 (Rv0652)	50S ribosomal protein L7/L12, RplL	13.4	7587-49	TrpdU	0.07	0.002
			7596-2	2NapdU	0.13	0.001
			12087-24	TrpdU	11.1	3.080
			14489-14	2NapdU	0.11	0.001
			14489-18	2NapdU	0.04	0.001
TPX (Rv1932)	Thiol peroxidase	15.1	7609-13	TrpdU	3.56	0.857
			14490-6	2NapdU	0.72	0.112
			14490-127	2NapdU	1.50	0.148

[1]These SOMAmer reagents are 48-50mer ssDNA oligonucleotides containing one type of modified nucleotide as indicated. The reagents are available at reasonable terms for researchers in the field under Global Access.
