# Supplementary material for: Potential of High-Affinity, Slow Off-Rate Modified Aptamer Reagents for Mycobacterium tuberculosis Proteins as Tools for Infection Models and Diagnostic Applications
Source: J Clin Microbiol. 2017 Sep 25;55(10):3072–88. doi: 10.1128/JCM.00469-17 (PMC5625393; doi:10.1128/JCM.00469-17)
Supplement: Supplemental material [file JCM.00469-17_zjm999095670s3.pdf]

TABLE S3 Characterization of SOMAmer reagents generated via SELEX with recombinant antigen 85 proteins. Cross-reactivity between the highly similar A85A, A85B, and A85C was determined in radiolabel equilibrium binding assays. Some SOMAmer reagents had been selected with antigen 85–fibronectin complexes and were tested for binding to free and complexed antigen 85 protein. Only SOMAmers that bound to free and complexed antigen 85 but not to fibronectin alone were further evaluated.

SOMAmer ID	Modified base	SELEX target	K_d (nM), affinity in equilibrium binding assay					
			A85A	A85A-FN	A85B	A85B-FN	A85C	A85C-FN
4948-1	NapdU	A85A	0.05		35		0.15	
4953-64	TrpdU	A85A	0.06		32		320	
12073-8	NapdU	A85A	0.09		72		320	
12073-32	NapdU	A85A	0.16		320		0.26	
12092-7	TrpdU	A85A	0.06		641		320	
14492-7	2NapdU	A85A-FN complex	0.24	0.21	200		320	
14492-11	2NapdU	A85A-FN complex	0.21	0.20	120		2.82	
14504-6	PPdU	A85A-FN complex	0.07	0.05	32		320	
4949-52	NapdU	A85B	0.91		6.39		14	
4954-5	TrpdU	A85B	0.15		0.31		320	
12074-5	NapdU	A85B	0.06		0.08		320	
12074-11	NapdU	A85B	0.12		0.14		13	
12093-26	TrpdU	A85B	6.32		0.26		320	
14493-5	2NapdU	A85B-FN complex	0.41		1.94	4.80	0.33	
14493-16	2NapdU	A85B-FN complex	0.22		0.29	0.84	2.00	
14505-57	PPdU	A85B-FN complex	0.46		0.13	0.28	1.83	
5569-2	2NapdU	A85C	11		30		0.01	
5575-1	PEdU	A85C	4.89		47		0.03	
4950-27	NapdU	A85C	320		212		0.03	
4955-49	TrpdU	A85C	320		320		0.26	
12075-16	NapdU	A85C	24		39		0.05	
12075-40	NapdU	A85C	320		37		0.13	
14494-53	2NapdU	A85C-FN complex	4.37		118		0.03	0.04
14494-124	2NapdU	A85C-FN complex	0.23		425		0.02	0.01
14506-48	PPdU	A85C-FN complex	12		97		0.04	0.04
14506-76	PPdU	A85C-FN complex	320		34		0.03	0.03
