# Supplementary material for: Potential of High-Affinity, Slow Off-Rate Modified Aptamer Reagents for Mycobacterium tuberculosis Proteins as Tools for Infection Models and Diagnostic Applications
Source: J Clin Microbiol. 2017 Sep 25;55(10):3072–88. doi: 10.1128/JCM.00469-17 (PMC5625393; doi:10.1128/JCM.00469-17)
Supplement: Supplemental material [file JCM.00469-17_zjm999095670s4.pdf]

TABLE S4 Effect of counter-selection with human serum on affinity and background of Mtb SOMAmer reagents. Standard SELEX applied counter-selection with albumin, casein, and prothrombin, while modified SELEX used 7.5% human serum in alternate SELEX rounds to eliminate cross-reactive or non-specific binding to human blood proteins. Apparent K_d and background RFU signals were obtained during SOMAmer validation via SOMAscan assay.

Mtb Protein (Gene)	Standard SELEX				SELEX with serum counter-selection			
	SeqID	Mod. base	$K_{d, app}$ (nM)	RFU_{bkgrnd}	SeqID	Mod. base	$K_{d, app}$ (nM)	RFU_{bkgrnd}
A85A (Rv3804c)	4953-64	TrpdU	0.018	6215	12092-7	TrpdU	0.007	981
A85B (Rv1886c)	4949-52	NapdU	0.018	3102	12074-5	NapdU	0.002	933
A85C (Rv0129c)	5569-2	2NapdU	0.005	1628	14494-124	2NapdU	0.006	377
GroES (Rv3418c)	7595-51	2NapdU	0.004	1375	14488-1	2NapdU	0.005	542
RL7 (Rv0652)	7596-2	2NapdU	0.001	1002	14489-14	2NapdU	0.001	522
MPT64 (Rv1980c)	7615-18	2NapdU	1.440	5102	14496-43	PPdU	0.028	700
Tpx (Rv1932)	7609-13	TrpdU	0.857	2418	14490-127	2NapdU	0.148	391
