# Supplementary material for: Potential of High-Affinity, Slow Off-Rate Modified Aptamer Reagents for Mycobacterium tuberculosis Proteins as Tools for Infection Models and Diagnostic Applications
Source: J Clin Microbiol. 2017 Sep 25;55(10):3072–88. doi: 10.1128/JCM.00469-17 (PMC5625393; doi:10.1128/JCM.00469-17)
Supplement: Supplemental material [file JCM.00469-17_zjm999095670s5.pdf]

TABLE S5 Demographic and clinical characteristics of the participants in this study. A. Serum samples tested on full SOMAscan (n=740). B. serum samples tested on TB SOMApanel (n=76, 67% male, median age 38 years).
C. Matching urine samples tested on TB SOMApanel (n=44, 66% male, median age 37 years).

A.

	TB neg HIV neg	TB neg HIV pos	TB pos HIV neg	TB pos HIV pos
Bangladesh	47	0	45	0
Colombia	36	3	25	6
Peru	46	12	40	16
South Africa	85	77	75	57
Uganda	0	0	10	10
Vietnam	45	17	41	22
Zimbabwe	3	15	1	6
Total	262	124	237	117
Male	117	60	133	65
Female	84	55	54	37
Sex unreported	61	9	50	15
Median Age	35	36	31	35
Age unreported	14	11	21	25

B.

	TB neg HIV neg	TB neg HIV pos	TB pos HIV neg	TB-pos HIV pos
Peru	2	3	7	2
South Africa	9	9	10	7
Vietnam	10	3	7	7
Total	21	15	24	16

C.

	TB neg HIV neg	TB neg HIV pos	TB pos HIV neg	TB-pos HIV pos
Peru	1	2	5	2
South Africa	1	6	4	7
Vietnam	4	2	5	5
Total	6	10	14	14
