# Supplementary material for: Potential of High-Affinity, Slow Off-Rate Modified Aptamer Reagents for Mycobacterium tuberculosis Proteins as Tools for Infection Models and Diagnostic Applications
Source: J Clin Microbiol. 2017 Sep 25;55(10):3072–88. doi: 10.1128/JCM.00469-17 (PMC5625393; doi:10.1128/JCM.00469-17)
Supplement: Supplemental material [file JCM.00469-17_zjm999095670s6.pdf]

TABLE S6 SOMAscan assay variations and sample pretreatment methods applied for optimization of signal-to-background ratio. Native Mtb proteins (CFPs) were spiked into buffer, serum, or urine, and clinical samples from TB and NTB were also tested to evaluate the benefit of individual optimization strategies.

Parameter	Standard Condition	Test Condition	Signal-to-background ratio
Equilibration time	3.5 h	16 h	Decreased
SOMAmer concentration	0.5 nM	2.5 nM	Decreased
Sample volume	50 µl	150 µl	Decreased
Non-specific competitor (Z-block)	20 µM	0.125-20 µM	Increased
NaCl	61.2 mM	500 mM	Decreased
$CaCl_2$	0	5 mM	Increased some
Tween-20 (PEG + fatty acid)	1.1%	0-1.1%	Slightly increased
Triton-X 100 (PEG + benzyl)	0	0-1%	Slightly increased
CHAPS (zwitterionic)	0	1%	Slightly increased
Heat pre-treatment	n/a	56°C, 30 min<br>80°C, 10 min	Slightly increased<br>N/A, sample gelling
Acid pre-treatment (to pH ~3.0)	n/a	Glycine/Tris, 10 min<br>Glycine/Tris, 30 min<br>HCl/NaOH, 10 min	Increased in serum<br>Increased in serum<br>Greatest increase in serum
